# Health services utilisation disparities between English speaking and non-English speaking background Australian infants

**DOI:** 10.1186/1471-2458-10-182

**Published:** 2010-04-08

**Authors:** Lixin Ou, Jack Chen, Ken Hillman

**Affiliations:** 1 Simpson Centre for Health Services Research, University of New South Wales, Sydney, Australia; 2 Sydney South West Area Health Service, Sydney, Australia

## Abstract

**Background:**

To examine the differences in health services utilisation and the associated risk factors between infants from non-English speaking background (NESB) and English speaking background (ESB) within Australia.

**Methods:**

We analysed data from a national representative longitudinal study, the Longitudinal Study of Australian Children (LSAC) which started in 2004. We used survey logistic regression coupled with survey multiple linear regression to examine the factors associated with health services utilisation.

**Results:**

Similar health status was observed between the two groups. In comparison to ESB infants, NESB infants were significantly less likely to use the following health services: maternal and child health centres or help lines (odds ratio [OR] 0.56; 95% confidence intervals [CI], 0.40-0.79); maternal and child health nurse visits (OR 0.68; 95% CI, 0.49-0.95); general practitioners (GPs) (OR 0.58; 95% CI, 0.40-0.83); and hospital outpatient clinics (OR 0.54; 95% CI, 0.31-0.93). Multivariate analysis results showed that the disparities could not be fully explained by the socioeconomic status and language barriers. The association between English proficiency and the service utilised was absent once the NESB was taken into account. Maternal characteristics, family size and income, private health insurance and region of residence were the key factors associated with health services utilisation.

**Conclusions:**

NESB infants accessed significantly less of the four most frequently used health services compared with ESB infants. Maternal characteristics and family socioeconomic status were linked to health services utilisation. The gaps in health services utilisation between NESB and ESB infants with regard to the use of maternal and child health centres or phone help, maternal and child health nurse visits, GPs and paediatricians require appropriate policy attentions and interventions.

## Background

An equitable use of health care amongst cultural and linguistic minority populations is one of the important goals within health care systems [1]. Prior work has addressed models for comprehensively understanding the equitable use of health care and for implementing effective interventions to reduce the disparities in health care [2,3]. In particular, Jacobs and colleagues concluded that language barriers have negative consequences for linguistic minorities in accessing health care [4].

Recent studies showed that the cultural and linguistic minority population has experienced more barriers in accessing health care due to socioeconomic disadvantages and limited English proficiency (LEP) [5-9]. The disparities raised concerns with respect to health services utilisation amongst children living with families from non-English speaking background (NESB). In the United States (US), children from NESB were less likely than those from English speaking background (ESB) to have a regular source of health care; were almost two and half times less likely as ESB children to see a specialist physician; and were less likely to use after-hours emergency care [10,11]. Similar evidence was also found in Canada and the United Kingdom (UK) [9,12,13].

In Australia, approximately 3.1 million people (15.6% of the population) spoke a language other than English at home [14]. The population with LEP has increased up to 10% since 2001 [14,15]. Moreover, people who mainly speak a language other than English have been considered a disadvantaged group with reduced access to government and community programs and services in Australia [16]. However, little is known about the disparities in health status and health services utilisation between NESB and ESB children in Australia. Given the different health care funding programs and systems amongst various English-speaking countries, as well as the composition of different minority ethnic groups in NESB populations, it is difficult to generalise the study findings of child health services utilisation from the US, Canada and the UK to the Australian context.

Numerous studies investigated language as a barrier in health services utilisation, and examined LEP as a predictor of access to health care [4,17,18]. However, other factors such as the familiarity with the health care system, cultural specific health beliefs and help-seeking behaviours that go beyond linguistic ability may also contribute to the disparities between minority ethnic groups and English-speaking populations [18]. Thus, it is important to understand how NESB plays its role in predicting the patterns of health services utilisation regardless of LEP.

Our study aimed to explore the disparities of health status and health services utilisation between NESB and ESB infants (3-18 months) in Australia, and to investigate the factors associated with the use of health services. We undertook this study using data from a large, Australian representative cohort study: the Longitudinal Study of Australian Children (LSAC) program [19]. The hypotheses of our study are that: 1) there are disparities in health status and health services utilisation between infants from NESB and ESB; 2) maternal characteristics, socioeconomic determinants and LEP are the main factors associated with the use of health services, and 3) both 'LEP' and 'NESB' can be independent predictors of health services utilisation.

## Methods

### Study design and sampling

We drew data from the first wave infant cohort (3-18 months old) of the LSAC. The detailed sampling design and its methodology have been described elsewhere [20]. Briefly, the first wave interviews of the LSAC were conducted between March and November 2004 with a two-stage stratified, clustered design. The sample frame of the LSAC was selected from the Health Insurance Commission (HIC) Medicare database. The sample elements were firstly stratified by state or territory and then by urban or rural status. Within each stratum, approximately one of the ten Australian postcodes was randomly included in the study as the primary sampling units to ensure proportional geographic representation. Only one child per family was recruited to the LSAC. Of 9259 infants selected by the HIC, 7951 families could be contacted as residents within those postcodes, and of these families, 5107 (64.2%) were recruited to the LSAC study.

### Data collection

A two-and-half-hour face-to-face interview was undertaken in the home by trained professional interviewers with the primary care-giving parent, mostly the biological mother (99.7%) but at times the biological father, step parent, adoptive parent, guardian, or someone who had a parental relationship to the child. The parents also completed a written questionnaire which was later returned. A brochure in nine languages which included information about this study was used. Apart from this brochure, an interpreter was used when required. Overall there were fifty languages involved. For each participating child, a written consent was obtained. The study was approved by the Australian Institute of Family Studies Ethics Committee.

The language background of infants was recorded by the interviewers using defined criteria [21]. NESB infants were defined as those whose parents speak a language other than English at home. A mother's English proficiency was assessed by asking the question, "how well do you consider the mother speak(s) English?" to the mother's partner or mother herself and coded by the interviewer with a four-point Likert scale (1 Very well; 2 Well; 3 Not well; 4 Not at all).

Indigenous infants (n = 230) were excluded from our study because they have been acknowledged as another minority group with health disadvantages, and the results were reported in a separate study [22].

### Health services utilisation measures

Health services utilisation was measured at three recognised service levels:

- Primary health care (maternal and child health centre or phone help, maternal and child health nurse visits, general practitioner (GP), hospital outpatient clinic, and other medical or dental services).

- Secondary health care (hospitalisation and hospital emergency wards).

- Tertiary health care (paediatrician and other specialist).

Respondents were asked to consider whether they used any of these services for the study child during the past 12 months. A hospitalisation in the present study was considered to be a hospital admission due to a medical condition or illness other than injury or accident.

### The predicting variables of health services utilisation

We used the Andersen health behaviour model to examine a wide range of variables in relation to health services utilisation [2]. The predisposing variables included infant characteristics (age, sex, and birth-weight), maternal characteristics (age, marital status, education status, employment status, proficiency in speaking English), and number of children within the household. Enabling variables were composed of family income per week, region of residence, and private health insurance. Health behaviour variables were smoking during pregnancy and drinking during pregnancy. We also included the advantage and disadvantage index of Socio-Economic Indexes for Areas (SEIFA) as an enabling variable. The SEIFA value is a composite measure from the 2001 census at the postcode of residence level, and a low value indicates an area of disadvantage [23]. We did not include health status as a need variable because health status in the baseline data can either be a cause or an outcome of health services utilisation [24].

### The concurrent health outcomes

Health outcomes were measured with current health status and physical outcome index. Health status used a 5-point Likert scale of the global health rating of infants by the surveyed parents (1 Excellent; 2 Very good; 3 Good; 4 Fair; 5 Poor). Physical outcome index is a composite score of the global health rating and 6-item special health care needs screening questions for infants [25].

### Data analyses

We analysed the data according to survey statistical principles and took into account the design features of the study. Analyses were weighted for the multistage sampling design, allowing for unequal probabilities of selection into the sample, and for no responses. First-order Taylor linearisation was used to obtain estimates of standard error taking account of the stratification and the correlation of responses within postcodes. Rao-Scott chi-square was used to examine the distributional difference between NESB and ESB infants for categorical variables and survey linear regression was carried out to test the mean difference of continuous variables between NESB and ESB infants. We also conducted survey logistic regression to examine the association between estimates of health services utilisation and some predictive variables. The total numbers included in the analyses were slightly varied due to missing values and non-responses to different items.

We used three models to explore the factors associated with health services utilisation in order to understand the independent predicting effects of NESB and LEP. The predictors of Model 1 included demographic variables of infants and their mothers, mothers' risk behaviours (smoking and drinking) during pregnancy, socioeconomic status (education, income), private insurance status, region of residence, NESB, as well as mothers' LEP. Model 2 differed from Model 1 only with the exclusion of NESB from the Model 1. Model 3 included a created composite variable from NESB and LEP with three categories: ESB, NESB with very well or well English proficiency, and NESB with not well or no English proficiency. Other predictors were the same for the three models. Model 1 examined the respective independent predicting effects for NESB and LEP after adjusting for other confounding variables. Model 2 explored the predictive power of LEP alone, while Model 3 examined the predicting power of both ethnicity and English proficiency in one single variable. Statistical significance was calculated with 95% confidence intervals [CIs]. All analyses were performed using Stata 9.2 (StataCorp. College Station, TX).

## Results

**Figure 1:**
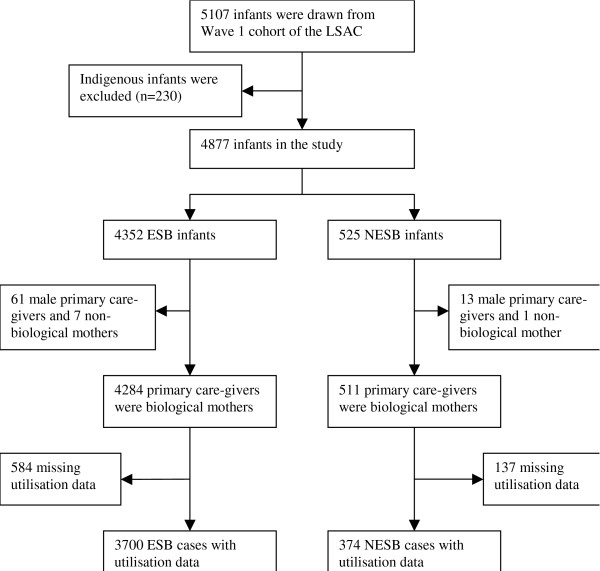
**Flowchart for data exclusion and numbers of infants included in the analyses**.

Figure 1 shows sample size changes at different stages in this study. To ensure valid comparisons of maternal characteristics, we included only those infants (4795) whose primary care-givers were their biological mothers (Figure 1). Of these, 511 (12%) were NESB infants. Health services utilisation data was available for 4074 infants consisting of 3700 ESB infants and 374 NESB infants. LEP data was available for 4007 cases. "Language other than English spoken at home" was available for 299 NESB infants. Only 3742 infants (3492 ESB and 250 NESB) were included in survey logistic regressions as a result of dropping cases with missing values. Within these models, the NESB group comprised 66 cases from European-NESB, 29 cases of Arabic-speaking, 20 cases of Vietnamese- speaking, 24 cases of Mandarin or Cantonese- speaking, 83 cases who spoke other languages, and 51 cases from NESB with unreported languages.

The primary languages other than English spoken at home by mothers were Arabic (2.4%), Vietnamese (1.7%), Cantonese/Mandarin (1.3%), Spanish (0.9%), Greek (0.9%), Italian (0.9%), German (0.6%), and others (6.3%). Close to 14% of NESB mothers kept confidential the language they spoke with their children. Amongst the most popular language groups, the majority of mothers spoke English very well or well (Arabic: 60.3%; Cantonese/Mandarin: 79.4%; Spanish, Greek, German and Italian: 100%), except for Vietnamese-speaking mothers (42.6%).

### Measures of health services utilisation

The average number of visits to services was significantly lower in NESB infants than in ESB infants for the previous 12 month period (2.6 vs 3.1, p < 0.001, Table 1). NESB infants were less likely to use four services: maternal and child health centres or phone help (p < 0.001), maternal and child nurse visits (p < 0.001), GPs (p < 0.001) and paediatricians (p < 0.001). No differences were observed in terms of hospital outpatient clinics, other medical and dental services, hospital emergency wards, hospitalisations and other specialists between the two groups. Amongst NESB infants, Vietnamese infants had the lowest level of access to the four services mentioned above, followed by Arabic, Chinese and other minority ethnic infants (Table 2). European NESB infants were at a similar level of services utilisation compared with ESB infants.

**Table 1:** Distribution of health services utilisation by language background and mothers' limited English proficiency (%) ^†^

Types of health services	Language background			Mothers' LEP^‡^			
		
	ESB^‡ ^(n = 3700)	NESB^‡ ^(n = 374)	p-value	Very well (n = 3826)	Well (n = 110)	Not well/not at all (n = 71)	p-value
Mean visits to any service below (± SE)	3.1(0.03)	2.6(0.10)	<0.001**	3.1 (0.03)	2.4 (0.18)	2.1 (0.22)	<0.001**
Maternal and child health centre or phone help	58.8	45.4	<0.001**	58.3	45.7	36.9	<0.001**
Maternal and child health nurse visits	68.3	55.2	<0.001**	68.1	55.5	44.6	<0.001**
General practitioner	82.4	69.9	<0.001**	82.1	69.8	55.2	<0.001**
Hospital outpatient clinic	9.8	7.2	0.15	9.6	9.2	8.6	0.94
Other medical or dental services	8.9	10.6	0.35	8.9	10.4	9.8	0.83
Hospital emergency ward	21.5	21	0.86	21.4	14.6	25.1	0.35
Hospitalisation	9.7	9.6	0.36	9.6	11.5	12.5	0.58
Paediatrician	39.3	25.9	<0.001**	39.2	18.1	9.9	<0.01**
Other specialist	12.2	11.8	0.83	12.2	7.1	10.7	0.29

^†^The proportions were weighted for survey data. ^‡ ^ESB = English speaking background; NESB = non-English speaking background, LEP = limited English proficiency.

** Significant at 1%.

**Table 2:** Distribution of health services utilisation by major ethnic groups of infants (%)^†^

Types of health services	ESB^‡^ (n = 3700)	European-NESB (n = 103)	Arabic (n = 49)	Vietnamese (n = 26)	Chinese (n = 38)	Others (n = 83)	Total (N = 3999)
Maternal and child health centre or phone help	58.8	57.4	40.4*	28.1**	42.0^§^	36.4**	57.4
Maternal and child health nurse visits	68.3	57.4*	55.2*	34.8**	62.6	52.5*	67.0
General practitioner	82.4	75.2	67.9*	47.9**	64.8*	75.8	81.3
Hospital outpatient clinic	9.8	5.3	2.1^§^	2.9	10.0	11.3	9.5
Other medical or dental services	8.9	15.4^§^	11.6	7.5	14.1	6.2	9.1
Hospital emergency ward	21.5	22.7	25.8	10.9	28.1	15.6	21.4
Hospitalisation	9.7	10.0	11.6	2.9	6.4	12.3	9.7
Pediatrician	39.3	41.1	16.6**	7.7**	17.8*	19.8**	37.9
Other specialist	12.2	10.7	8.0	9.0	18.2	11.1	12.1

^†^The proportions were weighted for survey data; ^‡^Reference Group.

* Significant at 5%; ** Significant at 1%. ^§^Marginally significant.

The decreasing order of the Mothers' LEP ('Very well' vs 'Well' vs 'Not well or Not at all') was associated with a trend of decreased use of the four services: maternal and child health centres or phone help (58.3% vs 45.7% vs 36.9%, p < 0.001), maternal and child nurse visits (68.1% vs 55.5% vs 44.6%, p < 0.001), GPs (82.1% vs 69.8% vs 55.2%, p < 0.001) and paediatricians (39.2% vs 18.1% vs 9.9%, p < 0.001).

### Infants, maternal, family and neighbourhood characteristics

There were no differences in age and sex distributions between the two groups, but significant lower birth weight was observed in NESB group (p < 0.001) (Table 3).

**Table 3:** Characteristics of the study children and their mothers by language speaking background ^†^

Characteristics	NESB^‡^ n = 511	ESB^§^ n = 4284	P-value
**Predisposing**			
Infant			
Mean age by month (SE)	8.9 (0.13)	8.8 (0.06)	0.65
Male, %	49.1	51.4	0.29
Mean birth weight(Gram)(SE)	3307.1(27.57)	3416.8(9.72)	<0.001**
Maternal			
Mothers' age groups, %			
15~20 years	2.4	3.8	0.03*
21~25 years	14.5	12.6	
26~30 years	29.8	26.1	
31~35 years	28.9	37.2	
36~40 years	20.7	16.6	
41 years or older	3.7	3.7	
Marital status, %			<0.001**
Married	85.6	72.1	
Single	14.4	27.9	
Education, %			0.23
Tertiary qualification^۴^	42.0	39.9	
Year 12 or equivalent only	25.7	23.4	
Under Year 12	32.3	36.8	
English proficiency, %			
Very well	51.8	98.1	<0.001**
Well	22.9	1.4	
Not well or not at all	25.4	0.6	
Mother employed, %	36.5	50.9	<0.001**
Smoking during pregnancy, %	8.4	17.6	<0.001**
Drinking during pregnancy, %	15.7	40.7	<0.001**
Number of children in household, %			
One	41.7	39.2	0.09
Two	32.9	37.4	
Three	15.4	16.4	
Four or more	10.0	7.1	
**Enabling**			
Family income per week, %			<0.001**
Less than $499	19.3	11.9	
$500-$999	38.0	32.7	
$1000-$1499	23.7	27.1	
$1500-$1999	11.2	14.2	
$2000 or more	7.9	14.0	
Mean SEIFA score (SE)^ץ^	994.8 (9.44)	1006.5 (4.20)	0.16
Region of residence, %			<0.001**
Metropolitan	93.6	64.2	
Non-metropolitan	6.4	35.8	
Private health insurance covered, %	27.9	47.8	<0.001**
**Infants' health status**			
Overall health rating for fair or poor, %	1.87	3.14	0.19
Physical outcome index (SE)	99.6(0.55)	100.2 (0.17)	0.27
Medical conditions or disabilities^¶^, %	5.1	5.3	0.85
Medical care needs due to injury or accident since birth, %	7.3	6.5	0.48

^† ^The percentages were weighted for the survey data. ^‡^NESB = non-English speaking background. ^§^ESB = English speaking. *Significant at 5%; **Significant at 1%.

^۴^Tertiary qualification includes postgraduate degree, graduate diploma/certificate, bachelor degree, and advanced diploma/diploma.

^ץ^SEIFA = Socio-Economic Index for Areas. ^¶^Study child has any medical conditions or disabilities that have lasted, or are likely to last, for six months or more.

Compared with ESB mothers, NESB mothers were more likely to be married, were less likely to be employed, and were less likely to smoke or drink alcohol during pregnancy (p < 0.001). Amongst NESB infants, 25.4% of infants were recorded with the mothers' LEP as not speaking English well or no English spoken at all. There were no significant differences in education levels between the two groups.

NESB families were less likely than ESB families to have a family income over $1000 per week (p < 0.001) and to have private health insurance (p < 0.001), but were more likely to live in the metropolitan area (p < 0.001). Neither group differed in the number of children in household and mean SEIFA scores. Health status measures were also comparable between the two groups (Table 3).

### Multivariate analyses: Model 1

Multivariate logistic regressions showed that NESB infants were less likely to utilise: maternal and child health centres or phone help (odds ratio [OR] 0.56; 95% CI, 0.40-0.79) (Table 4); maternal and child health nurse visits (OR 0.68; 95% CI, 0.49-0.95); GPs (OR 0.58; 95% CI, 0.40-0.83); and hospital outpatient clinics (OR 0.54; 95% CI, 0.31-0.93), after adjustment for infant and maternal characteristics, family socioeconomic status and LEP. Conversely, LEP was not significant for all models after adjusting for NESB.

**Table 4:** The survey logistic regression models (ORs and 95%CI) for health services utilisation and its associated risk factors (n = 3742)

Covariates	Maternal and child health centre or phone help	Maternal and child health nurse visits	General practitioner	Hospital outpatient clinic	Other medical or dental services
**Model 1^†^**					
NESB vs ESB infants^‡^	0.56 [0.40-0.79]**	0.68 [0.49-0.95]*	0.58 [0.40-0.83]**	0.54 [0.31-0.93]*	0.87 [0.48-1.55]
Infant sex					
male vs female	1.08 [0.93-1.24]	0.90 [0.78-1.02]	1.15 [0.94-1.41]	1.32 [1.06-1.65]*	1.16 [0.90-1.50]
Mothers' age					
15~20	1.0	1.0	1.0	1.0	1.0
21~25	1.65 [0.99-2.76]	1.48 [0.94-2.34]	1.29 [0.75-2.21]	1.09 [0.50-2.38]	1.92 [0.56-6.54]
26~30	2.12 [1.31-3.44]**	1.59 [0.98-2.57]	1.40 [0.84-2.34]	1.06 [0.51-2.20]	2.67 [0.72-9.96]
31~35	2.83 [1.74-4.61]**	1.50 [0.94-2.39]	1.55 [0.92-2.61]	1.26 [0.61-2.59]	2.56 [0.68-9.61]
36~40	2.83 [1.70-4.72]**	1.37 [0.85-2.19]	1.40 [0.79-2.48]	1.31 [0.61-2.82]	2.27 [0.58-8.87]
41 and older	1.90 [1.06-3.42]*	1.82 [1.02-3.26]*	1.70 [0.82-3.54]	1.66 [0.62-4.39]	3.19 [0.77-13.21]
Marital status					
married vs single	1.05 [0.87-1.28]	0.96 [0.79-1.18]	1.35 [1.07-1.70]*	0.67 [0.49-0.90]**	0.85 [0.62-1.17]
Mothers' education status					
under Year 12	1.0	1.0	1.0	1.0	1.0
year 12 or equivalent	1.22 [1.02-1.46]*	1.28 [1.03-1.59]*	1.11 [0.86-1.45]	1.30 [0.96-1.76]	0.82 [0.57-1.16]
tertiary qualification^۴^	1.78 [1.48-2.15]**	1.49 [1.22-1.84]**	1.23 [0.96-1.57]	1.40 [1.01-1.95]*	0.91 [0.67-1.22]
Mothers' LEP^‡^					
very well	1.0	1.0	1.0	1.0	1.0
well	0.99 [0.63-1.57]	0.71 [0.44-1.16]	0.71 [0.42-1.20]	1.54 [0.77-3.07]	1.10 [0.52-2.32]
not well/not at all	0.69 [0.32-1.47]	0.71 [0.33-1.54]	0.63 [0.30-1.34]	1.76 [0.56-5.49]	1.79 [0.73-4.36]
Smoking during pregnancy					
yes vs no	0.78 [0.63-0.96]*	0.91 [0.72-1.14]	1.04 [0.81-1.33]	1.05 [0.75-1.46]	1.07 [0.74-1.54]
Drink during pregnancy					
yes vs no	1.43 [1.22-1.67]**	1.13 [0.95-1.34]	1.23 [1.02-1.49]*	1.05 [0.81-1.35]	0.91 [0.71-1.18]
Number of children in household					
one	1.0	1.0	1.0	1.0	1.0
two	0.66 [0.55-0.77]**	0.91 [0.77-1.08]	0.98 [0.79-1.22]	0.76 [0.58-1.01]	1.10 [0.84-1.43]
three	0.46 [0.37-0.57]**	0.73 [0.59-0.91]**	0.98 [0.74-1.30]	0.79 [0.54-1.16]	1.65 [1.19-2.30]**
four or more	0.35 [0.26-0.48]**	0.58 [0.43-0.79]**	1.03 [0.72-1.48]	1.02 [0.63-1.65]	1.86 [1.20-2.87]**
Family income per week					
less than $499	1.0	1.0	1.0	1.0	1.0
$500 - $999	1.19 [0.91-1.55]	1.07 [0.80-1.42]	1.56 [1.19-2.05]**	1.25 [0.80-1.95]	0.98 [0.63-1.54]
$1000 - $1499	1.32 [1.02-1.70]*	1.02 [0.75-1.37]	1.66 [1.22-2.25]**	1.15 [0.71-1.86]	1.07 [0.65-1.75]
$1500 - $1999	1.18 [0.86-1.61]	1.32 [0.92-1.89]	1.63 [1.15-2.30]**	1.25 [0.76-2.06]	1.37 [0.81-2.32]
$2000 or more	1.42 [1.02-1.98]*	0.88 [0.61-1.28]	1.89 [1.30-2.73]**	1.23 [0.71-2.13]	1.54 [0.91-2.62]
Region of residence					
metropolitan vs non-metropolitan	1.14 [0.96-1.36]	1.10 [0.93-1.30]	1.46 [1.18-1.80]**	1.07 [0.79-1.45]	1.15 [0.87-1.51]
Private health insured					
yes vs no	1.04 [0.88-1.23]	1.00 [0.83-1.19]	0.99 [0.80-1.22]	0.69 [0.51-0.95]*	1.27 [0.93-1.72]
**Model 2**^§^					
Mothers' LEP					
very well	1.0	1.0	1.0	1.0	1.0
well	0.73 [0.46-1.17]	0.58 [0.37-0.92]*	0.53 [0.34-0.84]**	1.14 [0.57-2.27]	1.02 [0.50-2.07]
not well/not at all	0.46 [0.21-1.00]*	0.54 [0.26-1.14]	0.43 [0.21-0.88]*	1.15 [0.36-3.67]	1.61 [0.68-3.80]
**Model 3**^¶^					
NESB with very well or well English proficiency vs ESB	0.63 [0.46-0.86]**	0.58 [0.43-0.77]**	0.58 [0.42-0.79]**	0.58 [0.34-0.99]*	1.11 [0.68-1.83]
NESB with not well or no English proficiency vs ESB	0.21 [0.08-0.59]**	0.49 [0.21-1.14]	0.40 [0.18-0.87]*	1.38 [0.42-4.58]	1.80 [0.72-4.49]

**Covariates**	**Hospital emergency ward**	**Hospitalisation**	**Other specialist**	**Paediatrician**	

**Model 1**^†^					
NESB vs ESB infants^‡^	0.86 [0.57-1.28]	0.81 [0.46-1.43]	1.06 [0.66-1.70]	0.76 [0.52-1.10]	
Infant sex					
male vs female	1.05 [0.89-1.25]	1.22 [0.95-1.57]	1.19 [0.98-1.44]	1.23 [1.07-1.43]**	
Mothers' age					
15~20	1.0	1.0	1.0	1.0	
21~25	1.13 [0.65-1.96]	1.02 [0.53-1.96]	4.65 [0.99-21.77]	1.27 [0.69-2.33]	
26~30	1.01 [0.59-1.74]	0.86 [0.42-1.73]	7.80 [1.75-34.66]**	1.24 [0.66-2.32]	
31~35	1.08 [0.62-1.88]	0.86 [0.41-1.80]	7.54 [1.70-33.52]**	1.39 [0.75-2.57]	
36~40	1.04 [0.56-1.92]	0.95 [0.43-2.14]	9.92 [2.17-45.32]**	1.22 [0.65-2.30]	
41 and older	0.80 [0.40-1.60]	0.61 [0.22-1.73]	5.95 [1.18-30.01]*	1.51 [0.77-2.96]	
Marital status					
married vs single	0.83 [0.66-1.03]	0.91 [0.66-1.27]	0.88 [0.65-1.19]	0.98 [0.79-1.21]	
Mothers' Education					
under Year 12	1.0	1.0	1.0	1.0	
year 12 or equivalent	1.01 [0.81-1.26]	0.78 [0.58-1.07]	1.15 [0.85-1.55]	1.01 [0.81-1.26]	
tertiary qualification^۴^	1.01 [0.80-1.28]	0.87 [0.63-1.19]	1.23 [0.92-1.63]	1.29 [1.06-1.57]*	
Mothers' LEP^‡^					
very well	1.0	1.0	1.0	1.0	
well	0.71 [0.38-1.33]	1.29 [0.60-2.78]	0.63 [0.26-1.51]	0.56 [0.28-1.15]	
not well or not at all	1.69 [0.74-3.88]	1.13 [0.37-3.46]	1.44 [0.54-3.82]	0.50 [0.21-1.20]	
Smoking during pregnancy					
yes vs no	1.12 [0.90-1.41]	0.92 [0.66-1.28]	0.98 [0.70-1.37]	1.03 [0.82-1.29]	
Drinking during pregnancy					
yes vs no	0.92 [0.77-1.11]	0.95 [0.72-1.25]	1.14 [0.92-1.42]	1.04 [0.89-1.22]	
Number of children in household					
one	1.0	1.0	1.0	1.0	
two	1.01 [0.84-1.21]	1.07 [0.82-1.41]	0.80 [0.63-1.01]	0.95 [0.80-1.12]	
three	1.15 [0.87-1.51]	1.57 [1.12-2.20]**	0.86 [0.61-1.19]	0.80 [0.62-1.02]	
four or more	0.87 [0.59-1.28]	0.80 [0.45-1.43]	0.92 [0.58-1.46]	0.81 [0.58-1.13]	
Family income per week					
less than $499	1.0	1.0	1.0	1.0	
$500 - $999	1.02 [0.75-1.38]	1.05 [0.68-1.63]	0.87 [0.58-1.30]	1.03 [0.75-1.42]	
$1000 - $1499	1.05 [0.77-1.43]	0.86 [0.53-1.40]	1.08 [0.71-1.65]	0.90 [0.65-1.25]	
$1500 - $1999	0.98 [0.68-1.42]	0.93 [0.54-1.60]	0.81 [0.51-1.30]	1.00 [0.70-1.41]	
$2000 or more	1.16 [0.80-1.69]	0.94 [0.50-1.74]	0.88 [0.54-1.44]	1.02 [0.71-1.46]	
Region of residence					
metropolitan vs non- metropolitan	1.10 [0.89-1.35]	1.03 [0.79-1.34]	1.04 [0.83-1.30]	1.38 [1.13-1.68]**	
Private health insured					
yes vs no	0.90 [0.72-1.13]	0.96 [0.71-1.30]	1.64 [1.28-2.10]**	3.48 [2.93-4.13]**	
**Model 2**^§^					
Mothers' LEP					
very well	1.0	1.0	1.0	1.0	
well	0.65 [0.35-1.22]	1.15 [0.57-2.34]	0.65 [0.29-1.48]	0.49 [0.25-0.97]*	
not well or not at all	1.52 [0.64-3.56]	0.97 [0.34-2.77]	1.49 [0.59-3.80]	0.42 [0.17-1.01]	
**Model 3**					
NESB with very well or well English proficiency vs ESB	0.90 [0.63-1.28]	0.85 [0.50-1.43]	1.05 [0.69-1.58]	0.66 [0.48-0.91]*	
NESB with not well or no English proficiency vs ESB	1.79 [0.68-4.77]	0.66 [0.05-8.67]	1.36 [0.45-4.14]	0.54 [0.23-1.26]	

^† ^Model 1: The model included both NESB and mother's LEP as predictors, and all results were listed in the table.

^‡ ^NESB = non-English speaking background, ESB = English speaking background, LEP = limited English proficiency.

*Significant at 5%; **Significant at 1%.

^۴ ^Tertiary qualification includes postgraduate degree, graduate diploma/certificate, bachelor degree, and advanced diploma/diploma.

^§^Model 2: Removing variable NESB from Model 1. We only listed the results of mothers' LEP due to the similar results of other variables compared to Model 1.

^¶^Model 3: Combining the ethnicity and English proficiency into one single variable with three categories: ESB vs NESB with very well or well English proficiency; ESB vs NESB with not well or no English proficiency. Other variables were the same as that in Model 1. We only listed the results for the composite variable; and other results were similar to that in Model 1.

### Primary health care services

Younger mothers were significantly less likely to utilise maternal and child health centres or phone help alone. Married mothers were more likely than single mothers to see GPs for their infants (OR 1.35; 95% CI, 1.07-1.70), but were less likely to visit hospital outpatient clinics (OR 0.67; 95% CI, 0.49-0.90). Mothers with better education were more likely to use maternal and child health centres or phone help, maternal and child nurse visits, and hospital outpatient clinics for their infants. Those mothers who smoked during pregnancy were less likely to use maternal and child health centres or phone help, while those who drank alcohol during pregnancy were positively associated with the use of maternal and child health centres or phone help and GPs.

Mothers of infants who had three or more siblings in the household were least likely to use maternal and child health centres or phone help and maternal child nurse visits, but were most likely to see other medical or dental services for their infants. Positive correlations were observed between family income $2000 or more per week and visits to maternal and child health centres or phone help and GPs; and between living in metropolitan areas and visiting GPs. Those who were privately insured accessed less hospital outpatient clinics.

### Secondary health care services

There were no significant differences across all predicting variables in the use of hospital emergency wards. Similar results were also observed for hospital admissions except for the variable of 'number of children in the household'. Infants living in families with three children in the household were more likely to be admitted to hospital (OR 1.57; 95% CI, 1.12-2.20) in comparison with those infants from single-child families.

### Tertiary health care services

Infants whose mothers were aged 26 years or older and infants who were covered by private health insurance were more likely to visit other specialists. Male infants, infants whose mothers had tertiary qualifications, and infants living in metropolitan areas or having private health insurance were more likely to visit paediatricians.

### Multivariate analyses: Model 2

After removing NESB from Model 1, the results showed that those infants whose mothers spoke English very well were most likely to use maternal and child health centres or phone help, maternal and child health nurse visits, GPs, and paediatricians (Table 4).

### Multivariate analyses: Model 3

Model 3 showed that NESB infants were less likely than ESB infants to use maternal and child health centres or phone help, even when their mothers spoke English very well or well. Moreover, they also had reduced visits to maternal and child health nurses, GPs, hospital outpatient clinics, and paediatricians in comparison with ESB infants (Table 4). NESB mothers whose English proficiency was rated as not well or no English at all were least likely to use maternal and child health centres or phone help, and GPs for their infants.

## Discussion

To the best of our knowledge, this is the first study to examine the differences and determinants of health services utilisation between NESB and ESB infants at a national level in Australia. The study provides a comprehensive analysis across primary, secondary and tertiary health services utilisation amongst Australian infants. In contrast, the previous studies in Australia mostly focused on one particular health aspect or one set of health care characteristics, or on adult populations [26-28].

We found that NESB infants were disadvantaged in access to health services in Australia. Of the NESB infants in the study, the most disadvantaged were Vietnamese. Less than half of Vietnamese mothers were rated as performing very well or well in English proficiency and this may contribute to the lowest level of access to health services by Vietnamese infants, reflecting that LEP plays an important role in health services utilisation [17]. Interestingly, European NESB infants had comparable levels of health services utilisation to ESB infants. The results may be explained by their comparatively high level of English proficiency amongst European NESB mothers, as well as a similar cultural background to ESB mothers.

The results from multiple logistic regression analyses showed that there were significant disparities in a range of health services utilisation between NESB and ESB infants. Our results were consistent with previous findings regarding GP utilisation by minorities [12,29]. The findings indicated that the gaps in the use of primary health care, including maternal and child health centres or phone help, maternal and child health nurse visits, hospital outpatient clinics and GPs, were substantial between the two groups and in favour of ESB infants. Also consistent with previous finding [30], our results showed that these four types of health services were commonly used by infants during their first 12 months of life. Specific policies and interventions may be needed to target these areas in order to close the gap in infant health services utilisation.

Unlike the reported ethnic disparities in the use of hospital emergency wards and inpatient services in the UK [12,31], our study showed a similar attendance to hospital emergency wards and inpatient services between the two groups. There may be several possible reasons for such inappreciable disparities in the Australian setting. First, language barriers may not be crucial for disparities in access to hospital emergency wards and inpatients services in the Australian context. Second, the composition of NESB mothers in our study sample was different from that in other studies. Third, the majority of NESB mothers in our study spoke English very well or well in the most popular non-English languages groups (ie: Arabic, Cantonese/Mandarin, Spanish, Greek, German and Italian). Although 57% of Vietnamese-speaking mothers did not speak English well or not at all, other factors such as interpreting services in hospitals and strong family support may have mediated the impact of low language proficiency. Fourth, it is also possible that the lowest rates of service utilisation for the study infants during their first 12 months of life may have resulted in insufficient power to detect such a difference.

Amongst maternal, family and neighbourhood variables, mothers' age, education, family size and income, region of residence and private health insurance appeared to be useful predictors of utilisation rates. Our results concur with previous studies suggesting that maternal characteristics and family socioeconomic status play an important role in determining health care use amongst infants. It is reasonable that as primary care-givers, the mothers' knowledge of health and health care, as well as their help-seeking behaviour, to some extent, decide infants' health services utilisation patterns. In particular, well-educated mothers were more likely to use health care resources effectively and to make appropriate decisions in seeking health care for their infants [32-34].

The patterns of health services utilisation of families with three or more children were dramatically different from that of families with only one child. For example, families with three or more children accessed less maternal child health services but more other medical or dental services. We may speculate that the mothers who had three or more children had obtained adequate knowledge and experience from previous visits to maternal and child health services for the older siblings, thus reducing the need to visit for the study children. Other factors such as financial hardship, mothers' education levels and age may potentially affect the utilisation for large families in more complex ways. However, it appeared less clear why these mothers were more likely to use other medical or dental services. Further research is needed to explore the factors associated with health services utilisation for children with multiple siblings.

Our findings showed that socioeconomic disadvantages adversely affected children's health services utilisation, regardless of their family language background. Furthermore, our study provided detailed linkages between socioeconomic variables and each type of health services utilisation. For instance, children living in lower income households were less likely to utilise the services of maternal and child health centres or phone help and GPs. Children located in metropolitan area were more likely to visit GPs and paediatricians. Children who had private health insurance coverage were more likely to use paediatricians and other specialists, but were less likely to attend hospital outpatient clinics. These findings suggest that minimising the disparities in socioeconomic status may be an important way to achieve equitable access to health care for infants.

Our study showed that both the NESB variable and mothers' LEP have their unique positions in predicting health services utilisation. LEP has been a useful predictor for children's health and health care as it was proved to be a measure of linguistic capability [17]. Consistent with Flores and colleagues [17], we found that LEP was significantly associated with socioeconomic status such as family income and private health insurance when we examined the correlations between these variables separately. For example, mothers who had limited proficiency in speaking English were more likely than mothers who spoke English very well or well to have family income under $500 per week (32.4% vs 12.3%, p < 0.001), and were seven times as less likely to be covered by private health insurance (6.3% vs 46.7%, p < 0.001), which may indirectly affect the utilisation of some health services.

However, we found that LEP was not significantly associated with any types of health services utilisation when we examined NESB and LEP in the same model (Model 1), LEP was only significant when the NESB variable was removed from the model (Model 2). These findings suggest that the utilisation disparities between NESB and ESB infants cannot be solely explained by language barriers and measures of socioeconomic status as employed in the current study [35]. The culture, value, life style, social support, social capital, physical environment, wealth, health beliefs and help-seeking behaviours associated with diversified minority ethnic groups in Australia may also play important roles [3]. According to Australia government guideline since 2001 [36] NESB is no longer considered as an indicator for culture related disadvantage. Despite this, the combined results from Model 1 and Model 3 confirmed that NESB was still a powerful predictor of health services utilisation even amongst those NESB mothers who spoke English very well or well. This further indicates that other cultural factors may have a significant impact on the access to health care apart from socioeconomic status and LEP.

Our study had several limitations. Firstly, we only used the baseline data in providing the comparison of access to health care. Caution should be exercised in making causal inferences. Secondly, almost one in four of NESB mothers were rated as speaking English not well or not at all in our study compared with the Australian census (13.5%) [14]. It was not clear if our study was more representative than the census results given the possibility that those mothers with lower English proficiency may opt out of the census survey. Thirdly, our study response rate was low and data was based on self-report. Non-responses were mostly related to the low level of school completion of mothers (67.7% in current study vs 56.6% in the Australian census) as well as for mothers speaking a language other than English at home (15.0% vs 16.8% in the Australian census) [37]. We could not rule out the possibility that differential response patterns between NESB and ESB families may have introduced some selection biases. Moreover, 13.6% of ESB and 26.8% of NESB families did not report health services utilisation data. The highest levels of the missing data were observed in Arabic (27/76) and Vietnamese (22/48) groups. This may affect the assessment of factors associated with utilisation for NESB, in particular for Arabic and Vietnamese groups. However, the LSAC adopted a comprehensive strategy to identify the potential contributors to non-response and employed the calibration approach of Deville and Särndal [38] for adjustment on original design weight, the influences of non-response biases had been minimised within the LSAC sample [37]. Fourth, the socioeconomic status measures employed in the study may be limited. The complex relationships between different levels of socioeconomic status measures (i.e.: neighbourhood, community and individual level) and other important predictors such as family wealth, social support, social capital and health literacy were not explored in the current study. Caution needs to be exercised in interpreting the results [35].

On the other hand, our study also has several strengths. Our data were from a national representative sample and the measures of health services utilisation were comprehensive. We have also explored the differential predicting effects of NESB and LEP on health services utilisation, which may have important implications for other researchers. The results that NESB measure significantly predicted health services utilisation after adjusting for LEP and conventional socioeconomic status measures pointed to the great need in understanding the causal pathways that lead to disparities in health services utilisation between NESB and ESB infants.

## Conclusions

Amongst the nine types of health services, NESB infants were significantly less likely to access the four commonly used services: maternal and child health centres or phone help; maternal and child health nurse visits; GPs; and hospital outpatient clinics. Maternal characteristics, family size and income, private health insurance coverage and region of residence were the key factors associated with infants and their mothers' health services utilisation. Apart from language barriers and conventional socioeconomic status measures, there are more complex contributing factors towards disparities of health services utilisation between NESB and ESB infants and their mothers. Further research is needed to understand the complex causal pathways between ethnicity, LEP, social class, health services utilisation and health outcomes.

## Competing interests

The authors declare that they have no competing interests.

## Authors' contributions

LO has contributed to the conceptualisation, data analysis, interpretation of results, and the first draft of the manuscript. JC contributed to the conceptualisation, statistical analysis, interpretation of results, and preparation of final manuscript. KH contributed to the conceptualisation and preparation of the final manuscript. All authors read and approved the final manuscript.

## Pre-publication history

The pre-publication history for this paper can be accessed here:

http://www.biomedcentral.com/1471-2458/10/182/prepub
